# Microbiota-associated metabolic networks in gut-kidney communication and renal immune regulation: mechanisms and therapeutic potential

**DOI:** 10.3389/fmicb.2026.1899977

**Published:** 2026-08-27

**Authors:** Hongkun Zhang, Tao Zhang, Zhengchao Pan, Yue Zhang, Tingting Liang, Haijian Liu, Baisen Wang, Yongduo Yu, Shengzhi Wang

**Affiliations:** 1 Liaoning University of Traditional Chinese Medicine, Shenyang, China; 2 Second Affiliated Hospital, Liaoning University of Traditional Chinese Medicine, Shenyang, China; 3 Fourth Affiliated Hospital, Liaoning University of Traditional Chinese Medicine, Shenyang, China

**Keywords:** gut-kidney, immunometabolism, kidney diseases, metabolic-immune crosstalk, microbiota-associated metabolic networks, renal immune remodeling

## Abstract

Kidney disease development and progression involve not only local inflammation, immune dysregulation, and fibrosis but also alterations in gut microbiota composition and metabolic function. Microbiota-associated metabolic signals connect the intestinal ecosystem, host metabolism, and the renal immune microenvironment through their associations with intestinal barrier integrity, renal tubular epithelial homeostasis, immune cell function, and inflammatory-fibrotic responses. Depending on their biological origin, receptor engagement, target-cell specificity, and disease context, these signals may either support immune homeostasis and tissue repair or contribute to persistent inflammation and tissue remodeling. Representative mediators include short-chain fatty acids, tryptophan-derived metabolites, bile acid-related signaling molecules, indoxyl sulfate, p-cresyl sulfate, trimethylamine N-oxide, succinate, and other host-microbiota-associated metabolites. Their biological effects, however, cannot be interpreted independently of renal function because circulating metabolite levels are also influenced by impaired renal clearance, systemic inflammation, dietary factors, and host metabolism. Conversely, kidney dysfunction reshapes intestinal barrier integrity, microbial ecology, and metabolic output, establishing a dynamic gut-kidney metabolic-immune feedback network. In this Review, we summarize current evidence linking microbiota-associated metabolic networks to renal immune remodeling across different kidney diseases, highlighting metabolite origin, shared target-cell responses, disease-specific and context-dependent mechanisms, and current evidence limitations. We further discuss therapeutic strategies targeting this axis and emphasize key translational challenges, including distinguishing causal metabolic drivers from secondary metabolic alterations and developing precision interventions based on metabolic and immune phenotypes.

## 1 Introduction

Kidney diseases comprise a heterogeneous spectrum of disorders with diverse etiologies and immunopathological features, including acute kidney injury (AKI), chronic kidney disease (CKD), diabetic kidney disease (DKD), IgA nephropathy (IgAN), and lupus nephritis (LN) (Zhao et al., 2026; Rovin et al., 2021). Although these conditions differ substantially in their initiating factors and disease-specific pathogenic mechanisms, they frequently converge during disease progression on a series of shared pathological processes, including tubular epithelial injury, dysregulated immune activation, oxidative stress, impaired tissue repair, and progressive tubulointerstitial fibrosis (Li et al., 2021; Ohmori et al., 2026). Such pathological convergence does not imply identical molecular mechanisms across kidney diseases; rather, it suggests the existence of common metabolic-immune pathogenic modules that operate in a disease-dependent manner, with distinct upstream drivers, dominant signaling pathways, and temporal characteristics. Understanding how the systemic metabolic environment shapes local renal immune responses, while distinguishing shared regulatory programs from disease-specific mechanisms, is therefore essential for elucidating the pathogenesis and progression of kidney diseases.

Recent advances in metagenomics, metabolomics, and integrative multi-omics analyses have substantially expanded our understanding of the gut-kidney axis in renal diseases. Increasing evidence indicates that patients with kidney diseases exhibit not only alterations in gut microbial composition but also profound changes in microbial function and metabolic output (Lin et al., 2025). Acting as critical signaling mediators that connect the intestinal ecosystem, host metabolic status, and renal tissue responses, microbiota-associated metabolites participate in kidney disease progression by modulating intestinal barrier integrity, the systemic metabolic milieu, immune cell function, and the homeostasis of renal parenchymal cells (Liu et al., 2026; Kim et al., 2026). Under appropriate physiological or pathological conditions, short-chain fatty acids (SCFAs), selected tryptophan-derived metabolites, bile acid-related signaling molecules, and polyamines contribute to barrier maintenance, immune tolerance, and tissue repair. In contrast, host-microbiota co-metabolites such as indoxyl sulfate (IS), p-cresyl sulfate (PCS), and trimethylamine N-oxide (TMAO) have been shown in experimental settings to promote oxidative stress, inflammatory responses, and fibrotic remodeling (Qian and Wang, 2026; Hung et al., 2024; Nakagawa et al., 2021). Nevertheless, these metabolites cannot simply be classified as either protective or pathogenic. Their biological effects are highly context-dependent and are determined by multiple factors, including metabolic origin, local concentration, receptor availability, target-cell specificity, disease context, and renal functional status (Guan et al., 2026; Andrikopoulos et al., 2023).

Mechanistically, microbiota-associated metabolites do not function as isolated metabolite-receptor pairs. Instead, they collectively regulate multiple cellular compartments—including the intestinal barrier, renal tubular epithelial cells, podocytes, immune cells, and stromal cells—and ultimately converge on interconnected pathological processes such as altered thresholds of immune activation, metabolic stress, persistent inflammation, defective tissue repair, and fibrosis. In this Review, we define renal immune remodeling as the persistent reorganization of immune-cell composition and functional states, inflammatory signaling networks, immunometabolic programs, and immune-parenchymal cell communication during kidney disease progression. This process encompasses macrophage and dendritic cell reprogramming, disruption of T-cell immune homeostasis, immune stress responses in renal tubular epithelial cells, and the establishment of an inflammatory-fibrotic microenvironment. Diverse microbiota-derived metabolic signals regulate immune-cell function, tubular injury responses, and tissue repair through signaling pathways involving the aryl hydrocarbon receptor (AHR), farnesoid X receptor (FXR)/Takeda G protein-coupled receptor 5 (TGR5), G protein-coupled receptors (GPCRs), nuclear factor-κB (NF-κB), the NLRP3 inflammasome, transforming growth factor-*β* (TGF-β)/Smad signaling, and hypoxia-inducible factor-1α (HIF-1α) (Tian et al., 2023; Foresto-Neto et al., 2024; Zhou et al., 2024). Although these pathways converge on several common downstream pathological outcomes, their relative contributions and biological consequences remain highly dependent on cell type and disease context.

Importantly, metabolic-immune communication along the gut-kidney axis is intrinsically bidirectional. Gut microbial communities and their metabolic products shape systemic inflammation and renal immune responses, whereas declining renal function, the uremic milieu, systemic inflammation, dietary alterations, and therapeutic interventions reciprocally impair intestinal barrier integrity and reshape microbial ecology, substrate utilization, and metabolic activity. Consequently, elevated circulating concentrations of specific metabolites may reflect enhanced microbial production, impaired renal clearance, or both. Microbiota-associated metabolic abnormalities should therefore not be interpreted as unidirectional drivers of kidney injury but rather as components of a dynamic feedback network integrating microbial metabolism, host metabolic regulation, renal clearance, and tissue responses.

Despite growing interest in microbiota-associated metabolites in kidney diseases, the current evidence remains highly uneven. First, metabolites differ substantially in their biological origin and mechanistic significance. Direct microbial metabolites, host-microbiota co-metabolites, metabolites produced by both microbial and host pathways, and predominantly host-derived immunometabolic mediators should not be considered mechanistically equivalent. Second, the strongest mechanistic evidence currently comes from studies of AKI, CKD, DKD, uremic toxin accumulation, and experimental renal fibrosis. By contrast, investigations of IgAN and LN have primarily focused on associations among gut microbial composition, metabolic profiles, and immune phenotypes, whereas direct mechanistic evidence linking specific microbiota-derived metabolites to renal immune regulation remains relatively limited. Furthermore, observational studies in humans, experimental animal models, *in vitro* investigations, and mechanistic evidence derived from non-renal diseases each support different levels of inference. Associations observed in clinical studies or mechanistic extrapolations from other disease contexts should therefore not be interpreted as definitive evidence of causal mechanisms in kidney diseases.

In this Review, we conceptualize the microbiota-associated metabolic network as a dynamic regulatory system linking alterations in the intestinal ecosystem, host metabolic status, renal function, and renal immune remodeling. The Review is organized around five major themes: the biological origins of microbiota-associated metabolic signals, their regulation of renal target cells and shared immunopathological modules, kidney-to-gut feedback mechanisms, disease context-dependent metabolic-immune interactions, and emerging therapeutic strategies. We first summarize how metabolite classes of distinct biological origins regulate renal target cells and immune pathways, followed by a discussion of how kidney injury reciprocally reshapes intestinal barrier function, microbial ecology, and metabolic output. We then examine the dynamic roles of metabolic-immune interactions during kidney injury, chronic inflammation, and fibrotic remodeling, together with their therapeutic implications. Finally, we distinguish metabolic-immune processes shared across multiple kidney diseases from disease-specific pathways and clarify the interpretive boundaries among evidence derived from human studies, experimental kidney disease models, and indirect mechanistic investigations.

## 2 Literature search strategy

To comprehensively summarize the mechanistic roles and therapeutic implications of microbiota-associated metabolic networks in mediating gut-kidney communication during kidney diseases, we conducted a literature search of the PubMed and Web of Science databases. The search included studies published up to May 2026 and employed combinations of keywords related to the gut-kidney axis, microbial metabolism, immune regulation, and kidney diseases. During the revision process, selected additional relevant publications published after the initial search period were incorporated to update the evidence base and address reviewer comments, including “gut-kidney axis,” “gut microbiota,” “microbial metabolites,” “short-chain fatty acids,” “tryptophan metabolites,” “bile acids,” “uremic toxins,” “immune regulation,” “inflammation,” “fibrosis,” “acute kidney injury,” “chronic kidney disease,” “diabetic kidney disease,” “IgA nephropathy,” and “lupus nephritis.”

This Review adopts a mechanism-oriented narrative approach. Eligible studies included investigations of microbial metabolite production, host-microbiota metabolic interactions, immune regulation, and the pathogenesis and progression of kidney diseases. Evidence was drawn from human observational studies, longitudinal cohort studies, animal models, *in vitro* experiments, and mechanistic investigations. To elucidate broadly conserved metabolic-immune regulatory mechanisms, selected mechanistic studies from inflammatory, metabolic, and immune-mediated diseases outside the field of nephrology were also considered. These findings were interpreted cautiously and only when supported by kidney disease-related evidence.

To avoid conflating association with causation, the available evidence was interpreted according to study design and level of mechanistic support. Human studies were primarily used to evaluate clinical associations between microbiota-associated metabolic alterations and disease phenotypes. Longitudinal cohort studies and interventional investigations provided additional evidence regarding temporal relationships and potential causal links. Experimental animal models were considered the principal source of evidence for determining the functional roles of specific metabolites or signaling pathways in renal injury, inflammation, and fibrotic remodeling, whereas *in vitro* studies were used primarily to elucidate molecular mechanisms and target-cell responses. Mechanistic evidence derived from non-renal diseases was included only as complementary support for proposing potentially conserved regulatory networks, and its limitations for extrapolation to kidney diseases are explicitly acknowledged throughout this Review.

It should also be emphasized that circulating concentrations of certain metabolites may be substantially influenced by renal function itself. For example, blood levels of uremic toxin-related metabolites reflect not only microbial production but also renal clearance, including glomerular filtration, tubular secretion, protein binding, as well as dietary factors, pharmacological interventions, and dialysis. Accordingly, when interpreting associations between microbiota-associated metabolic alterations and kidney diseases, we carefully considered the reciprocal effects of kidney dysfunction and distinguished putative disease-driving metabolic signals from secondary metabolic changes resulting from impaired renal function.

Rather than providing a systematic review or quantitative evidence synthesis, this Review aims to establish a conceptual framework for microbiota-associated metabolic network-mediated renal immune remodeling. Particular emphasis is placed on critically evaluating the biological origins, mechanistic actions, evidence hierarchy, and interpretive boundaries of distinct microbiota-associated metabolic signals within the context of kidney diseases.

## 3 Molecular mechanisms linking microbiota-associated metabolic signals to renal immunity

The gut-kidney axis represents a dynamic and integrated network shaped by microbial metabolism, host metabolic regulation, renal functional status, and immune responses. In this Review, the term microbiota-associated metabolic network refers to the interconnected system in which metabolite-derived signals from distinct biological origins interact through shared target cells, convergent signaling pathways, competition for metabolic substrates, and bidirectional gut-kidney feedback. Rather than acting independently, diverse metabolites collectively influence renal tubular epithelial cells, podocytes, macrophages, lymphocytes, and fibroblasts, with their downstream effects converging on signaling pathways involving nuclear factor-κB (NF-κB), the NLRP3 inflammasome, the aryl hydrocarbon receptor (AHR), hypoxia-inducible factor-1α (HIF-1α), mitochondrial homeostasis, epigenetic regulation, and transforming growth factor-*β* (TGF-β)-mediated fibrotic signaling.

This network exhibits regulatory properties that extend beyond individual metabolite-receptor interactions. Metabolites with similar biological functions may preserve epithelial barrier integrity, immune tolerance, or tissue repair through distinct receptors and signaling pathways, thereby providing a degree of functional redundancy. Consequently, impairment of one protective metabolic pathway may be partially compensated by adaptive changes in other metabolic axes. Conversely, activation of the same signaling pathway may produce divergent—or even opposing—biological outcomes depending on ligand identity, target-cell state, and disease context.

At the systems level, these metabolic interactions ultimately converge on several interconnected pathological modules, including disruption of intestinal barrier function, stress responses and defective repair in renal tubular epithelial cells, immune-cell reprogramming, persistent inflammatory amplification, and progressive fibrotic remodeling. Although these pathological modules recur across multiple kidney diseases, the dominant metabolites, modes of interaction, and supporting evidence differ substantially according to disease context. Guided by this framework, the following sections first summarize microbiota-associated metabolic signals according to their biological origins and renal effects, and then integrate their network organization from the perspectives of shared target cells, convergent signaling pathways, and reciprocal feedback regulation. Finally, we discuss how kidney injury itself reshapes the intestinal microenvironment and microbial metabolic output.

### 3.1 Regulation of renal immunity by microbiota-associated metabolic signals classified according to biological origin

The metabolic signals involved in gut-kidney communication are not exclusively generated by the gut microbiota but instead arise from microbial metabolism, host metabolism, and their continuous metabolic interplay. Based on biological origin and the nature of host-microbiota metabolic interactions, microbiota-associated metabolic signals discussed in this Review are categorized into four groups: direct microbial metabolites, host-microbiota co-metabolites, metabolites produced by both microbial and host pathways, and predominantly host-derived immunometabolic mediators whose production or activity is influenced by the gut microbiota (Table 1). This classification is intended to distinguish the biological origins and relative microbial contributions of individual metabolites rather than to imply equivalent degrees of microbial dependence across these four categories.

**Table 1:** Origin-based classification of microbiota-associated metabolic signals involved in gut-kidney metabolic-immune interactions: mechanisms, evidence basis, and major limitations.

Origin category	Representative metabolite	Primary source	Major target cells	Key signaling pathways	Role in kidney diseases	Study types	Evidence basis and main limitations	References
Direct microbial metabolite	Acetate	Microbial fermentation of dietary carbohydrates	Renal tubular epithelial cells, podocytes, macrophages	Histone acetylation-miR-493-3p/MIF; GPR43-AMPKα	Suppresses macrophage infiltration and calcium oxalate-induced kidney injury; excessive GPR43 activation may exacerbate podocyte insulin resistance in DKD	Animal models, cell studies, and human tissue analyses	Kidney-specific experimental and tissue evidence supports context-dependent effects; the direction of action differs according to disease model, receptor activation, and target cell type	(Zhu et al., 2023; Lu et al., 2021)
Direct microbial metabolite	Propionate	Microbial fermentation of dietary substrates	Renal tubular epithelial cells, Treg cells	Transcriptional remodeling; Treg-associated immune regulation	Attenuates inflammation and fibrosis during AKI-to-CKD transition and may promote Treg expansion in patients with ESRD	Animal studies, cell studies, and small human studies	Functional evidence is mainly derived from experimental kidney injury models; available human studies are small and do not establish effects on long-term renal clinical outcomes	(Corte-Iglesias et al., 2024; Anft et al., 2024)
Direct microbial metabolite	Butyrate	Fermentation of dietary fiber by butyrate-producing bacteria	Renal tubular epithelial cells, mesangial cells	HDAC2-ULK1; AMPK-SIRT1-PGC-1α	Enhances autophagy and mitochondrial homeostasis, reducing inflammation, oxidative injury, and fibrosis in DKD	Experimental DKD models and cell studies	Mechanistic support is derived predominantly from exogenous butyrate administration in animal and cell models; direct human intervention evidence in kidney disease is lacking	(Ma and Wang, 2022; Ye et al., 2024)
Direct microbial metabolite	Indole derivatives, primarily IPA	Microbial tryptophan metabolism	Podocytes, renal tubular epithelial cells, immune cells	SIRT1-mitochondrial homeostasis; AHR-NF-κB	Improves mitochondrial function in DKD but may aggravate inflammation and fibrosis in cisplatin-associated CKD models	Animal studies, cell studies, and patient samples	Kidney-specific studies demonstrate ligand- and disease-dependent effects; the biological direction varies with metabolite identity, dose, injury context, and AHR signaling status	(Zeng et al., 2025; Wang Q. et al., 2025)
Host-microbiota co-metabolite	Secondary bile acids and receptor ligands	Microbial transformation of primary bile acids	Renal tubular epithelial cells, podocytes, interstitial cells	TGR5/FXR; TGF-β-SMAD3-TAZ	Regulates renal metabolism and fibrosis; selected receptor agonists improve DKD and kidney organoid fibrosis	Animal studies, cell studies, and kidney organoids	Evidence is primarily preclinical and supports receptor-related metabolic and antifibrotic effects; individual bile acid species and receptor ligands have distinct actions and should not be interpreted as a uniform pathway	(Zhou et al., 2025; Yang et al., 2024)
Host-microbiota co-metabolite	Indoxyl sulfate (IS)	Microbial indole production followed by host sulfation	Renal tubular epithelial cells, macrophages, fibroblasts	Inflammatory priming, cellular senescence, and profibrotic signaling	Promotes low-grade inflammation, tubular senescence, and interstitial fibrosis	Gene-deficient animal models and cell studies	Functional kidney-specific evidence supports inflammatory, senescence-associated, and profibrotic effects; circulating concentrations are strongly influenced by renal clearance, protein binding, diet, and treatment exposure	(Hou et al., 2023; Ribeiro et al., 2023)
Host-microbiota co-metabolite	p-Cresyl sulfate (PCS)	Microbial p-cresol production followed by host sulfation	Renal tubular epithelial cells, endothelial cells	OAT1/OAT3; oxidative stress and apoptosis	Induces tubular injury, although recent studies have not demonstrated aggravation of ischemic AKI	Animal and cell studies	Experimental evidence remains relatively limited and findings are not fully consistent across models; circulating concentrations are also influenced by impaired renal elimination	(Falconi et al., 2023; Wei et al., 2022)
Host-microbiota co-metabolite	Trimethylamine N-oxide (TMAO)	Microbial TMA production followed by hepatic FMO3 oxidation	Renal tubular epithelial cells, macrophages, fibroblasts	NLRP3; lactate-p300/H4K12 lactylation	Promotes renal inflammation, profibrotic macrophage polarization, and CKD progression	Animal studies, cell studies, and patient tissue analyses	Multiple kidney-related experimental studies support inflammatory and profibrotic effects; however, circulating concentrations are affected by diet, hepatic metabolism, renal clearance, and underlying kidney function	(Tang et al., 2026; Fang et al., 2021)
Produced by both host and microbiota	Polyamines, including spermine and spermidine	Diet, host metabolism, and gut microbial metabolism	Renal tubular epithelial cells, macrophages	Nrf2; SMOX-spermine-ATG5 autophagy	Spermidine suppresses fibrosis, whereas spermine depletion promotes cellular senescence and kidney injury	Animal studies, cell studies, and genetic intervention studies	Experimental studies support roles in autophagy, oxidative stress, senescence, and fibrosis; biological effects depend on the specific polyamine, tissue concentration, and relative host versus microbial contribution	(Aihara et al., 2023; Luo et al., 2024)
Produced by both host and microbiota	Lactate	Microbial fermentation and host glycolysis	Renal tubular epithelial cells, macrophages, fibroblasts	PFKFB3-lactylation-NF-κB; H3K14la-KLF5	Promotes tubular phenotypic transition, inflammatory transcription, and renal fibrosis	Human tissue analyses, animal studies, and cell studies	Kidney-specific tissue and experimental studies support lactylation-related inflammatory and fibrotic mechanisms; intrarenal lactate is predominantly host-derived, and the direct microbial contribution remains unclear	(Wang et al., 2024; Zhang et al., 2024)
Produced by both host and microbiota	Succinate	Microbial metabolism and the host tricarboxylic acid cycle	Renal tubular epithelial cells, macrophages	SUCNR1; apoptosis and profibrotic macrophage activation	Induces tubular apoptosis and promotes macrophage-mediated interstitial fibrosis	Animal and cell studies	Experimental evidence supports SUCNR1-related tubular injury and macrophage-mediated fibrosis; the proportion of kidney-relevant succinate directly derived from gut microbiota has not been established	(Tao et al., 2025; Pu et al., 2023)
Predominantly host-derived immunometabolite	Kynurenine	Host IDO1/TDO-mediated tryptophan metabolism	Renal tubular epithelial cells, cyst-lining epithelial cells, immune cells	HIF-IDO1-kynurenine axis	Mediates hypoxic preconditioning-associated renoprotection but may also promote progression of polycystic kidney disease	AKI and PKD animal models and cell studies	Kidney-specific experimental evidence demonstrates opposing effects across disease models; the pathway is predominantly host-derived, and its relationship with gut microbiota is indirect and context dependent	(Torosyan et al., 2021; Nguyen et al., 2023)
Predominantly host-derived immunometabolite	ATP/adenosine	ATP released from injured cells and converted to adenosine by CD39/CD73	Renal tubular epithelial cells, macrophages, T cells	ATP-P2X7-NLRP3; adenosine-A2A receptor signaling	ATP amplifies inflammation and fibrosis, whereas A2A receptor signaling limits progressive kidney injury	Gene-knockout models and experimental nephritis models	Experimental kidney-disease studies support opposing inflammatory effects of ATP and adenosine signaling; these metabolites are primarily host-derived, and direct human evidence remains limited	(Qian et al., 2024; Truong et al., 2016)

Despite their distinct origins, these metabolic signals often act on overlapping cellular targets, including renal tubular epithelial cells, podocytes, immune cells, and stromal cells. However, the disease contexts, levels of supporting evidence, and the strength of mechanistic inference vary considerably among different metabolic pathways. Some metabolic axes are supported by convergent evidence from experimental kidney disease models, *in vitro* mechanistic studies, genetic or pharmacological interventions, and human clinical investigations. Others are supported primarily by disease-associated metabolomic alterations or by mechanistic evidence derived from non-renal diseases, and therefore provide indirect rather than definitive evidence for their roles in kidney disease pathogenesis.

Overall, metabolic signals of distinct biological origins do not act independently through isolated pathways but instead converge at the levels of shared target cells and signaling nodes. Renal tubular epithelial cells represent major effector cells that integrate diverse metabolic inputs. These cells respond to SCFAs, indole-derived metabolites, IS, PCS, lactate, succinate, and purinergic signals, resulting in alterations in mitochondrial homeostasis, autophagy, oxidative stress, cellular senescence, and injury-repair programs (Corte-Iglesias et al., 2024; Ma and Wang, 2022; Ye et al., 2024; Zeng et al., 2025; Wang Q. et al., 2025; Hou et al., 2023; Ribeiro et al., 2023; Falconi et al., 2023; Wei et al., 2022; Wang et al., 2024; Zhang et al., 2024; Tao et al., 2025; Pu et al., 2023; Torosyan et al., 2021; Nguyen et al., 2023; Qian et al., 2024; Truong et al., 2016; Favero et al., 2024). Macrophages constitute another important metabolic sensing hub. Signals mediated by acetate, propionate, TMAO, lactate, succinate, and polyamines can regulate macrophage inflammatory states and profibrotic functions through G protein-coupled receptors, the NLRP3 inflammasome, succinate receptor 1 (SUCNR1), oxidative stress, and epigenetic mechanisms (Zhu et al., 2023; Corte-Iglesias et al., 2024; Tang et al., 2026; Fang et al., 2021; Aihara et al., 2023; Luo et al., 2024; Wang et al., 2024; Zhang et al., 2024; Tao et al., 2025; Pu et al., 2023). Moreover, the parallel actions of metabolic signals on tubular epithelial cells, immune cells, and fibroblasts may collectively contribute to persistent inflammation, defective tissue repair, and tubulointerstitial fibrosis through intercellular communication (Hou et al., 2023; Ribeiro et al., 2023; Tang et al., 2026; Fang et al., 2021; Wang et al., 2024; Zhang et al., 2024; Tao et al., 2025; Pu et al., 2023).

Available evidence further suggests that different metabolic signals may exhibit functional synergy, antagonism, partial redundancy, or compensatory changes. SCFAs, selected indole derivatives, bile acid receptor signaling, and polyamines can support tissue protection through distinct effects on mitochondrial function, autophagy, antioxidant responses, and immune homeostasis (Corte-Iglesias et al., 2024; Anft et al., 2024; Ma and Wang, 2022; Ye et al., 2024; Zeng et al., 2025; Wang Q. et al., 2025; Zhou et al., 2025; Yang et al., 2024; Aihara et al., 2023; Luo et al., 2024). These findings imply that several protective metabolic pathways may partially overlap in function. However, direct evidence for functional redundancy or compensatory activity remains limited, as few studies have simultaneously manipulated multiple metabolic axes within the same kidney disease model. By contrast, IS, PCS, TMAO, lactate, and succinate have been associated with oxidative stress, inflammasome activation, cellular senescence, immunometabolic reprogramming, and profibrotic responses (Hou et al., 2023; Ribeiro et al., 2023; Falconi et al., 2023; Wei et al., 2022; Tang et al., 2026; Fang et al., 2021; Wang et al., 2024; Zhang et al., 2024; Tao et al., 2025; Pu et al., 2023). When acting on common target cells, these injury-associated signals may exert additive inflammatory and fibrotic effects, although such interactions remain to be verified through combined multi-metabolite intervention studies.

Several metabolic axes also display pronounced directionality and disease context dependence. Indole-3-propionic acid (IPA) exerts protective effects in experimental DKD by preserving sirtuin 1 (SIRT1) activity and mitochondrial homeostasis, whereas in cisplatin-associated CKD it may aggravate inflammation and fibrosis through AHR-NF-κB signaling. These contrasting effects indicate that AHR-mediated responses are shaped by ligand properties, the nature of renal injury, and the local tissue environment (Zeng et al., 2025; Wang Q. et al., 2025). Acetate can attenuate hyperoxaluria-associated kidney injury, whereas excessive activation of GPR43 may worsen podocyte insulin resistance in DKD (Zhu et al., 2023; Lu et al., 2021). The kynurenine pathway may contribute to protection in hypoxic preconditioning-associated AKI but may also promote the progression of polycystic kidney disease (Torosyan et al., 2021; Nguyen et al., 2023). Similarly, ATP and adenosine exert opposing immunoregulatory effects through distinct signaling pathways, including P2X7-NLRP3 and adenosine A2A receptor signaling (Qian et al., 2024; Truong et al., 2016). The overall biological output of the metabolic network is therefore more likely to depend on the relative composition of metabolites, their local concentrations, receptor-expression patterns, and the disease microenvironment than on an isolated increase or decrease in any single metabolite.

These network-level interactions are further shaped by feedback from renal functional status and the disease environment. Declining kidney function not only reduces the clearance of selected circulating metabolites but also alters intestinal substrate availability, microbial ecology, and metabolic output, thereby increasing the burden of uremia-associated metabolites while impairing certain protective metabolic functions (Shi et al., 2024; Lauriola et al., 2026; Qu et al., 2022; Liu W. et al., 2025; Gunasekaran Rajalakshmi et al., 2026; Zhang et al., 2023; Lee et al., 2026; Hsu and Tain, 2026). Persistent inflammation, oxidative stress, and fibrosis may in turn further modify host metabolism and the response thresholds of local renal cells (Qu et al., 2022; Liu W. et al., 2025; Hsu and Tain, 2026). Accordingly, elevated circulating levels of IS, PCS, and TMAO may reflect increased microbial production or host conversion, but they may also predominantly result from impaired renal clearance, dietary factors, pharmacological exposure, or other secondary influences. Few studies have simultaneously quantified multiple metabolites, metabolic fluxes, and renal clearance processes. Direct evidence for functional redundancy, compensatory reorganization, and signaling synergy therefore remains limited, and the proposed network architecture is currently inferred primarily from shared target cells, convergent signaling nodes, and bidirectional gut-kidney feedback.

The distribution of evidence across different kidney diseases is also markedly uneven. Studies of SCFAs have mainly focused on the AKI-to-CKD transition, DKD, and other forms of experimental kidney injury, with support from animal models, cellular studies, and small-scale human investigations (Zhu et al., 2023; Lu et al., 2021; Corte-Iglesias et al., 2024; Anft et al., 2024; Ma and Wang, 2022; Ye et al., 2024). Indole-derived metabolites exert divergent effects in experimental DKD and cisplatin-associated CKD, further illustrating the ligand- and disease context-dependent nature of AHR signaling (Zeng et al., 2025; Wang Q. et al., 2025). Evidence for bile acid-FXR/TGR5 signaling is derived predominantly from DKD animal models and kidney organoid studies (Zhou et al., 2025; Yang et al., 2024). The proinflammatory, cytotoxic, and profibrotic effects of IS, PCS, and TMAO are supported mainly by animal and cellular studies of CKD, DKD, and experimental renal fibrosis, together with evidence from selected patient tissue samples (Hou et al., 2023; Ribeiro et al., 2023; Falconi et al., 2023; Wei et al., 2022; Tang et al., 2026; Fang et al., 2021). Lactate, succinate, polyamines, kynurenine, and ATP/adenosine have also been implicated experimentally in renal inflammation, metabolic stress, and fibrosis. However, for several of these mediators, the relative contribution of microbial production and their direct relationship with the gut microbiota remain insufficiently defined (Aihara et al., 2023; Luo et al., 2024; Wang et al., 2024; Zhang et al., 2024; Tao et al., 2025; Pu et al., 2023; Torosyan et al., 2021; Nguyen et al., 2023; Qian et al., 2024; Truong et al., 2016).

By comparison, direct mechanistic studies of specific metabolic signals or integrated metabolic networks remain limited in IgAN and LN. Overall, the strongest functional evidence is derived from experimental kidney disease models, genetic or pharmacological interventions, and *in vitro* studies, whereas human investigations more commonly support associations between metabolic profiles and disease phenotypes. The available evidence therefore supports the concept that multiple metabolic signals contribute to renal immune remodeling through shared target cells, convergent pathways, and reciprocal feedback regulation. Nevertheless, it remains insufficient to fully reconstruct the architecture, temporal organization, and causal direction of microbiota-associated metabolic networks across different kidney diseases.

Feedback regulation is a defining feature that distinguishes microbiota-associated metabolic networks from unidirectional metabolite pathways. Kidney disease can itself reshape the intestinal ecosystem and its metabolic functions through declining renal function, systemic inflammation, altered substrate availability, and therapeutic exposure (Shi et al., 2024). As kidney function deteriorates, the accumulation of uremia-associated metabolites, heightened inflammatory activity, and disruption of systemic metabolic homeostasis may collectively impair intestinal barrier integrity, alter microbial ecological niches, and modify substrate utilization. These changes may further reduce selected protective metabolic outputs while enhancing pathways involved in the generation of uremic toxin precursors (Lauriola et al., 2026). Such reciprocal remodeling can establish self-reinforcing loops or compensatory reorganization between intestinal metabolic dysfunction and kidney injury, rather than reflecting a simple unidirectional effect of gut-derived factors on the kidney.

Following the decline in renal function, retained uremia-associated metabolites can induce stress responses in intestinal epithelial cells, disrupt tight junction protein expression, and compromise barrier stability (Qu et al., 2022). In parallel, proinflammatory mediators generated by local renal and systemic inflammation, including tumor necrosis factor-*α* (TNF-α), interleukin-1β (IL-1β), and interferon-*γ* (IFN-γ), may act on the intestinal mucosa through the circulation and alter epithelial function, mucosal immune activity, and microenvironmental homeostasis (Liu W. et al., 2025). Impaired barrier function may increase intestinal permeability and reshape the ecological conditions governing microbial colonization and competition. This process may weaken selected protective metabolic pathways while further favoring metabolic programs associated with uremic toxin production.

Beyond barrier disruption, disease-associated changes in the systemic metabolic environment may directly influence microbial function. Evidence from metagenomic, transcriptomic, and metabolomic studies supports this concept. Several cohort studies of patients with CKD have shown that declining kidney function is accompanied by functional remodeling of the gut microbiota, including reduced pathways related to SCFA production, enhanced aromatic amino acid metabolism, and increased functional capacity for the generation of uremic toxins (Gunasekaran Rajalakshmi et al., 2026; Zhang et al., 2023). Renal dysfunction also alters the intestinal availability of protein-derived metabolites, nitrogen-containing substrates, and other nutrients, thereby reshaping microbial substrate preferences and metabolic outputs (Lee et al., 2026).

At the same time, chronic inflammation, oxidative stress, and changes in the redox environment of the intestinal lumen may compromise the stability of obligate anaerobes, particularly selected SCFA-producing bacteria (Hsu and Tain, 2026). Kidney disease may therefore be accompanied by reduced SCFA production, disrupted bile acid transformation, and remodeling of tryptophan metabolic networks. However, the direction and magnitude of these changes are likely to vary according to disease type, stage of renal impairment, dietary exposure, and individual host characteristics.

Therapeutic interventions may further contribute to remodeling of the gut-kidney axis. Immunosuppressive agents, glucocorticoids, antibiotics, and dietary modifications can alter inflammatory activity, microbial composition, and substrate availability, thereby influencing microbial metabolic profiles (Lee et al., 2024). Consequently, microbiota and metabolite abnormalities observed in patients may reflect a composite of disease-related mechanisms, secondary adaptations to declining renal function, and treatment-associated effects. They should not therefore be uniformly interpreted as primary drivers of disease initiation.

Taken together, direct human evidence for kidney injury-driven remodeling of the intestinal ecosystem is currently concentrated mainly in CKD. Existing studies support associations between declining renal function, impaired intestinal barrier integrity, microbial functional reorganization, and enhanced production of uremic toxin precursors. However, the temporal sequence and independent causal contribution of these changes remain incompletely defined, and findings from CKD cannot be directly generalized to all kidney diseases. Future studies integrating longitudinal cohorts, analyses matched for renal function, source tracing of circulating metabolites, and functional intervention experiments will be essential to distinguish metabolic pathways that actively contribute to disease progression from secondary alterations caused by renal impairment or therapeutic exposure.

## 4 Dynamic evolution of the microbial metabolic-immune network during kidney disease progression

The contribution of microbial metabolic-immune networks to kidney disease is strongly dependent on both temporal stage and disease context. Current evidence does not support a uniform linear trajectory shared by all kidney diseases. Instead, several recurring patterns of metabolic-immune change appear to emerge under specific pathological conditions. Studies of AKI have mainly focused on how changes in the metabolic environment influence immune activation, tubular injury, and tissue repair during acute damage. Research in CKD and experimental renal fibrosis has placed greater emphasis on declining renal function, accumulation of uremia-associated metabolites, chronic inflammation, and fibrotic feedback. In DKD, these processes are additionally shaped by hyperglycemia, lipotoxicity, and systemic metabolic dysfunction. By comparison, direct evidence defining the temporal dynamics of metabolic-immune networks in IgAN and LN remains limited.

**Figure 1:**
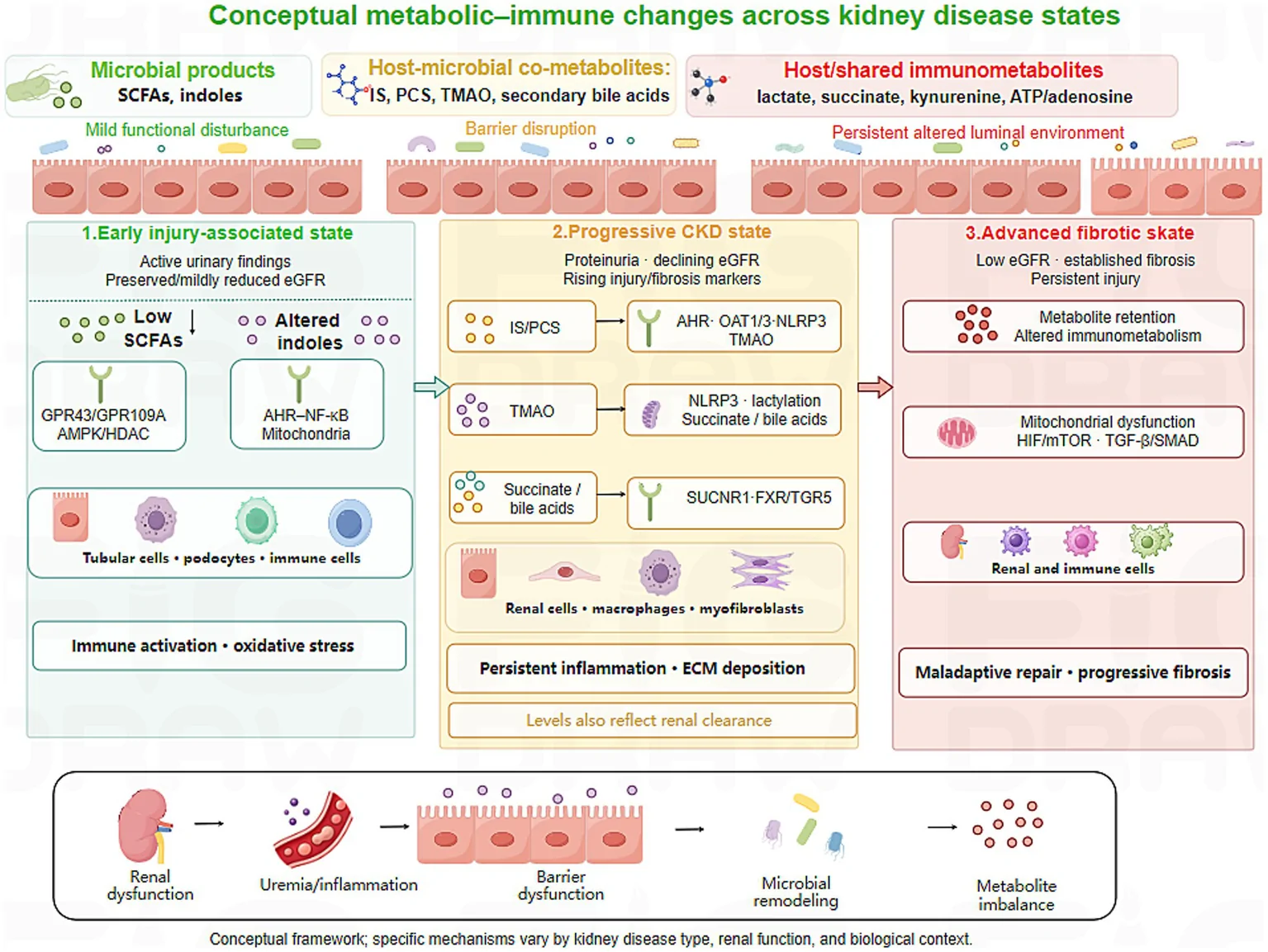
Dynamic remodeling of the gut-kidney metabolic-immune axis across representative kidney disease states. This figure presents a conceptual framework illustrating how microbiota-associated metabolic signals contribute to renal immune remodeling under different kidney disease contexts. According to their biological origins, these signals include direct microbial metabolites, host-microbiota co-metabolites, metabolites produced by both the host and microbiota, and predominantly host-derived immunometabolites influenced by the gut microbiota. Representative metabolic alterations converge on shared renal target cells and signaling pathways, including renal tubular epithelial cells, podocytes, immune cells, and fibroblasts, thereby influencing metabolic stress, immune activation, tissue repair, persistent inflammation, and fibrotic remodeling. The framework also highlights the reverse effects of kidney dysfunction, uremia, and systemic inflammation on intestinal barrier integrity, microbial ecology, and metabolic output. The illustrated states represent conceptual disease-associated contexts rather than universal clinical stages, and substantial overlap may exist among different kidney diseases.

Accordingly, this section does not propose a universal stage-based model applicable to all kidney diseases. Instead, based on the available evidence, we separately discuss representative metabolic-immune alterations associated with acute kidney injury, chronic loss of renal function and fibrosis, and prolonged disease states. The mechanisms summarized in each subsection should therefore be interpreted in relation to the specific disease context, experimental model, and level of supporting evidence. The principal patterns of change are illustrated in Figure 1.

### 4.1 Altered metabolic environments and disruption of immune homeostasis in acute kidney injury

AKI may be triggered by ischemia, infection, toxic exposure, or other acute insults and is characterized by tubular injury, local immune activation, and systemic metabolic disturbance. During this process, the functional activity of the gut microbiota and the profile of microbiota-associated metabolites may also change (Ren et al., 2024; Li J. et al., 2026). Evidence from animal models and selected clinical metabolomic studies suggests that AKI is accompanied by reduced SCFA-associated metabolism, disrupted energy metabolism, and increased inflammatory metabolic signaling (Wang et al., 2026; Yang et al., 2026). These alterations may impair the ability of renal tissues to adapt to acute stress and may influence both local immune responses and subsequent tissue repair.

The available evidence more strongly supports a role for microbiota-associated metabolic abnormalities as modifiers of immune responses during AKI than as isolated initiators of disease. Loss of protective metabolic signals may weaken antioxidant defenses, metabolic homeostasis, and repair capacity in renal tubular epithelial cells. It may also impair regulatory immune-cell activity, thereby lowering the threshold for amplification of local inflammation (Yu et al., 2023; Dalga et al., 2025). In parallel, damage-associated molecular patterns released from acutely injured tissues promote the recruitment and activation of neutrophils, macrophages, and other inflammatory cells, further reshaping the renal immune microenvironment (Lv et al., 2021).

Animal studies have shown that modifying gut microbial composition or the intestinal metabolic environment can alter the severity of inflammation, tissue damage, and renal functional recovery in experimental AKI, suggesting that gut-derived metabolic signals participate in immune regulation after acute renal injury (Cao et al., 2025; Liu et al., 2021; Xie et al., 2025). However, human evidence remains largely associative and is based primarily on relationships between metabolomic profiles and disease severity. It therefore remains uncertain whether microbiota-associated metabolic changes directly determine the onset, severity, or recovery trajectory of AKI.

### 4.2 Metabolic-immune feedback during chronic loss of renal function and fibrosis

During CKD progression and experimental renal fibrosis, microbiota-associated metabolic abnormalities may gradually become integrated into self-sustaining networks of chronic inflammation and tissue remodeling. Human cohort and multi-omics studies indicate that CKD progression is frequently accompanied by loss of protective microbial functions, increased concentrations of uremia-associated metabolites, and disruption of host-microbiota co-metabolism (Hellman et al., 2025; Wang H. et al., 2023; Laiola et al., 2025). These changes coexist with chronic low-grade inflammation, increased oxidative stress, and immune-cell dysfunction, although their temporal sequence and independent pathogenic contributions require cautious interpretation (Wang et al., 2020).

Persistent metabolic stress may impair tubular epithelial repair and drive a transition from adaptive regeneration toward incomplete or maladaptive repair (Mao et al., 2026). Single-cell transcriptomic and spatial-omics studies have further revealed complex communication networks among tubular epithelial cells, macrophages, fibroblasts, and other immune and stromal populations in CKD. Within this cellular ecosystem, an abnormal metabolic environment may help sustain proinflammatory and profibrotic cell states (Xu et al., 2025; Li L. et al., 2026). Persistent immune activation, defective parenchymal repair, and stromal-cell responses can therefore reinforce one another and ultimately promote tubulointerstitial fibrosis (Aggarwal et al., 2024).

In this setting, the gut-kidney axis may evolve into a relatively stable feedback loop. Declining renal function restricts metabolite clearance, compromises intestinal barrier integrity, and reshapes microbial ecology. In turn, abnormal microbial metabolic outputs may intensify systemic inflammation and local renal immune activation, thereby further promoting tissue injury (Jin et al., 2026; Mihajlovic et al., 2021). Nevertheless, elevations in circulating metabolites may in some cases primarily reflect impaired renal excretion rather than increased microbial production. Their independent contributions to CKD progression therefore require further validation through analyses matched for renal function, source-tracing studies, genetic approaches, metabolite-specific interventions, and long-term longitudinal follow-up.

### 4.3 Persistent metabolic alterations and potential immune susceptibility during long-term kidney disease

During the course of chronic kidney disease, stabilization of conventional measures of renal function does not necessarily indicate complete restoration of the gut-kidney metabolic-immune network. Nevertheless, longitudinal evidence demonstrating persistent alterations in microbial function and metabolic profiles during clinically stable phases of kidney disease remains limited. Longitudinal studies in metabolic liver disease and inflammatory bowel disease have shown that gut microbial and metabolic signatures may persist despite changes in clinical status or may be associated with subsequent disease relapse (Wu et al., 2024; Peters, 2026). However, these observations provide only indirect support for the concept of long-term metabolic remodeling and cannot be assumed to occur in kidney diseases.

From a biological perspective, sustained metabolic disturbances may alter the responsiveness of renal tubular epithelial cells and immune cells through immunometabolic regulation, epigenetic modification, and cellular reprogramming. For example, reduced SCFA availability has been linked to altered histone modifications and changes in the expression of genes involved in immune regulation (Jiang et al., 2025). Likewise, persistent oxidative stress and an abnormal metabolic milieu may modify the activation thresholds and functional states of renal tubular epithelial cells, macrophages, and lymphocytes (Tao et al., 2026). These findings raise the possibility that previous metabolic exposures may continue to influence cellular behavior and tissue responsiveness even after the initial injurious stimuli have diminished.

The concept of metabolic memory provides a useful framework for describing these potential long-lasting effects. However, in the context of kidney disease, it should currently be regarded as a hypothesis requiring further validation rather than as an established pathogenic mechanism. Direct evidence supporting metabolic memory in renal disease remains limited, and much of its theoretical basis has been extrapolated from other chronic metabolic disorders or from indirect mechanistic studies. Accordingly, persistent metabolic abnormalities should not at present be interpreted as having a definitive causal role in disease progression. Future investigations integrating longitudinal multi-omics profiling, cell type-specific metabolic tracing, recovery-phase disease models, and targeted metabolic interventions will be required to determine whether previous metabolic perturbations continue to influence cellular function, disease recurrence, or therapeutic responsiveness after renal function or inflammatory activity has improved.

Recent advances in single-cell multi-omics, spatial transcriptomics, and spatial metabolomics provide powerful new approaches for dissecting the relationship between metabolic abnormalities and cell-state organization in long-term kidney disease. Integrating metabolic signals with cellular functional states and longitudinal clinical trajectories may help distinguish metabolic mechanisms that actively contribute to disease persistence or recurrence from secondary alterations that simply reflect chronic tissue injury.

## 5 Therapeutic strategies targeting microbiota-associated metabolic networks and renal immune interactions: translational perspectives

**Figure 2:**
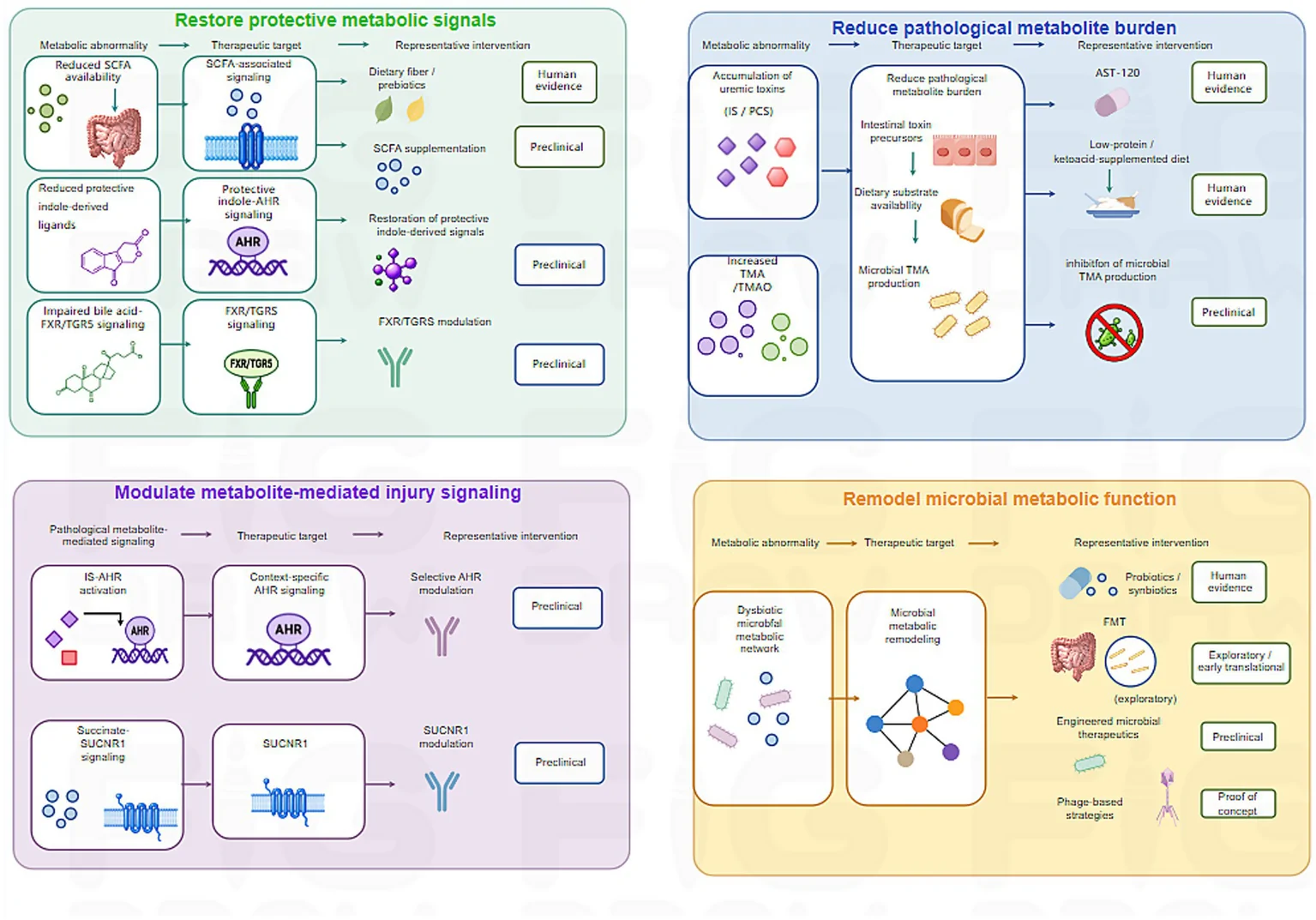
Therapeutic framework targeting microbiota-associated metabolic networks in kidney diseases. This framework summarizes four therapeutic directions corresponding to Section 5: Restoration of protective metabolic signals, reduction of pathological metabolite burden, modulation of metabolite-mediated injury signaling, and remodeling of microbial metabolic function. The restoration module incorporates three major protective metabolic axes, including SCFAs, selected indole-derived signals, and bile acid-FXR/TGR5 signaling, while distinguishing protective indole-AHR signaling from pathological AHR activation. The framework further separates upstream reduction of harmful metabolite exposure from downstream modulation of injury-related signaling pathways, including IS-AHR and succinate-SUCNR1 pathways. Representative interventions are annotated according to evidence maturity (human evidence, preclinical, exploratory/early translational, and proof-of-concept). Patient stratification, monitoring strategies, and translational limitations are discussed separately in Section 5.4 and Table 2.

Advances in research on the gut-kidney axis have increasingly identified microbiota-associated metabolic dysregulation as a potentially modifiable therapeutic target. Nevertheless, most intervention strategies remain at the stage of proof-of-concept studies, preclinical evaluation, or early-phase clinical investigation. Rather than indiscriminately enhancing or suppressing individual metabolic pathways, therapeutic approaches targeting the interaction between microbiota-associated metabolic networks and renal immunity aim to restore protective metabolic functions, reduce pathological metabolic burden, limit maladaptive downstream signaling, and improve microbial metabolic output in a disease-specific manner. The principal intervention strategies are summarized in Figure 2 and Table 2.

**Table 2:** Therapeutic strategies targeting microbiota-associated metabolic networks in kidney diseases: mechanisms, current evidence, and translational challenges.

Intervention direction	Representative strategy	Principal mechanism	Evidence and potential benefits in kidney diseases	Stage of development	Major limitations	References
Restoration of metabolite function	Dietary fiber/prebiotics	Increase fermentable substrate availability and promote SCFA production	Small RCTs in CKD have reported increased propionate levels and modulation of selected microbial or inflammatory markers	Small clinical trials	Small sample sizes, short intervention periods, and inconsistent findings	(Nogueira-Rio et al., 2025; Biruete et al., 2021)
Restoration of metabolite function	SCFA supplementation	Activate GPR43/GPR109A signaling and suppress oxidative stress and NF-κB activation	Attenuates proteinuria, inflammation, and tissue injury in experimental models of diabetic kidney disease	Preclinical	Lack of interventional evidence in patients with CKD	(Li et al., 2020; Huang et al., 2020)
Modulation of tryptophan metabolism	Indole ligands/AHR modulation	Regulate mitochondrial function, inflammation, and oxidative stress through PKA- or AHR-dependent signaling	Indole-3-aldehyde attenuates cisplatin-induced AKI, whereas inhibition of pathological AHR signaling improves experimental membranous nephropathy	Animal and in vitro studies	AHR effects are highly ligand- and disease-context dependent	(Yuan et al., 2024; Wang Y. N. et al., 2023)
Modulation of bile acid signaling	FXR/TGR5 agonism	Improve mitochondrial metabolism and suppress lipotoxicity and fibrotic signaling	Restoration of TGR5 signaling or dual FXR/TGR5 agonism attenuates diabetic and obesity-related kidney injury	Preclinical	Lack of kidney disease-specific clinical trials	(Lin et al., 2023; Wang et al., 2018)
Reduction of uremic toxin burden	AST-120	Adsorb gut-derived uremic toxin precursors and reduce the accumulation of IS and related metabolites	Modulates microbial function in CKD; animal studies suggest improvements in intestinal barrier integrity and kidney injury	Animal studies and small human studies	Effects on clinically meaningful renal outcomes remain inconsistent	(Hsu et al., 2022; Yoshifuji et al., 2018)
Reduction of uremic toxin substrates	Low-protein diet with ketoanalogues	Reduce aromatic amino acid availability and protein fermentation	May alter the gut microbiota and reduce selected uremic toxins, although findings remain inconsistent	Clinical studies	Risk of malnutrition and limited long-term adherence	(Rocchetti et al., 2021; Wu et al., 2020)
Inhibition of pathological metabolite production	TMA lyase inhibitors	Inhibit microbial CutC/CutD activity and reduce TMAO production	Iodomethylcholine reduces tubulointerstitial fibrosis and improves renal function in mice	Preclinical	No human validation and unknown long-term safety	(Gupta et al., 2020; Cheng et al., 2025)
Blockade of metabolite-responsive receptors	AHR/SUCNR1 inhibition	Block profibrotic IS-AHR or succinate-SUCNR1 signaling	AHR deletion attenuates renal aging and fibrosis, whereas SUCNR1 antagonism suppresses high-glucose-induced epithelial-mesenchymal transition	Animal and *in vitro* studies	Evidence for SUCNR1 inhibition is derived mainly from in vitro studies	(Xie et al., 2024; Zhang et al., 2024)
Remodeling of microbial function	Probiotics/synbiotics	Increase beneficial microbial populations, strengthen barrier function, and reduce uremic toxin production	Small RCTs have reported reductions in selected uremic toxins or inflammatory markers	Small clinical trials	Marked strain-specific variability and inconsistent renal functional benefits	(Mitrović et al., 2023; Kuskunov et al., 2023)
Remodeling of microbial function	Fecal microbiota transplantation (FMT)	Reconstruct microbial communities and amino acid metabolic networks	CKD animal studies have reported reduced PCS levels or delayed kidney injury, although some studies found no improvement in renal function	Animal studies	Lack of human CKD trials and standardized protocols	(Barba et al., 2020; Arteaga-Muller et al., 2024)
Precision microbiota remodeling	Engineered bacteria/synthetic microbial consortia	Selectively consume uremic toxin precursors or reprogram microbial metabolic pathways	SynCKD and engineered *Escherichia coli* reduce uremic toxin production and ameliorate kidney injury in animal models	Preclinical	Lack of human efficacy data and long-term colonization evidence in CKD	(Beau et al., 2025; Lambert et al., 2023)
Precision microbial depletion	Bacteriophage therapy	Selectively eliminate target bacteria and reshape microbial metabolic networks	Targeted modulation of the microbiota and metabolome has been achieved in non-renal disease models	Proof of concept in non-renal models	No direct evidence of therapeutic efficacy in CKD	(Zhang et al., 2025; Liu C. et al., 2025)
Precision therapeutic strategy	Multi-omics-based patient stratification	Integrate genetic, single-cell, and spatial-omics data to identify disease subtypes and therapeutic targets	Can identify cell–cell networks and potential targets involved in renal fibrosis, but has not yet been shown to guide microbiota-targeted treatment	Cohort and mechanistic studies	Not yet validated as a treatment-selection strategy	(Abedini et al., 2024; Liu H. et al., 2022)

Current approaches can be broadly classified into four categories: restoring protective metabolic signals, reducing the burden of pathogenic metabolites, blocking metabolite-associated injurious pathways, and remodeling microbial metabolic function. These strategies differ substantially in both the strength of supporting evidence and their degree of translational maturity. Dietary fiber supplementation, prebiotics, low-protein or ketoacid-supplemented diets, AST-120, and selected probiotics or synbiotics have progressed to human studies, although most clinical investigations have focused on changes in microbial composition, circulating metabolites, or inflammatory biomarkers rather than hard renal outcomes (Nogueira-Rio et al., 2025; Biruete et al., 2021; Hsu et al., 2022; Rocchetti et al., 2021; Wu et al., 2020; Mitrović et al., 2023; Kuskunov et al., 2023). In contrast, supplementation with SCFAs, modulation of AHR or succinate receptor 1 (SUCNR1), activation of farnesoid X receptor (FXR) or Takeda G protein-coupled receptor 5 (TGR5), and inhibition of trimethylamine (TMA) production are currently supported predominantly by experimental animal and *in vitro* studies (Li et al., 2020; Huang et al., 2020; Yuan et al., 2024; Wang Y. N. et al., 2023; Lin et al., 2023; Wang et al., 2018; Gupta et al., 2020; Cheng et al., 2025; Xie et al., 2024; Zhang et al., 2024). Other strategies, including fecal microbiota transplantation (FMT), engineered microbial therapeutics, and bacteriophage-based approaches, remain largely at the stage of animal studies or proof-of-concept investigations conducted outside the field of kidney disease (Barba et al., 2020; Arteaga-Muller et al., 2024; Beau et al., 2025; Lambert et al., 2023; Zhang et al., 2025; Liu C. et al., 2025). Consequently, evaluation of their clinical potential should be guided by study design, disease context, and kidney-specific clinical outcomes rather than by mechanistic plausibility alone.

### 5.1 Restoring protective metabolic signals

Reduced production of protective metabolites represents a characteristic feature of gut-kidney axis dysfunction in several kidney diseases. Under experimental conditions, SCFAs, selected indole-derived metabolites, and bile acid receptor signaling have been shown to influence intestinal barrier integrity, mitochondrial function, immune-cell phenotypes, and tissue repair, making restoration of these pathways an attractive therapeutic strategy (Corte-Iglesias et al., 2024; Anft et al., 2024; Ma and Wang, 2022; Ye et al., 2024; Zeng et al., 2025; Wang Q. et al., 2025; Zhou et al., 2025; Yang et al., 2024; Li et al., 2020; Huang et al., 2020; Yuan et al., 2024; Wang Y. N. et al., 2023; Lin et al., 2023; Wang et al., 2018). However, the biological effects of these signals are highly dependent on dose, receptor availability, target-cell specificity, and disease context, and therefore should not be regarded as universally protective pathways across all kidney diseases.

Enhancing endogenous SCFA production is currently among the best-studied strategies in human populations. Dietary fiber and prebiotics increase the availability of fermentable substrates for the gut microbiota and have been reported to modify SCFA levels, microbial composition, and inflammatory biomarkers in selected CKD studies (Nogueira-Rio et al., 2025; Biruete et al., 2021; Wathanavasin et al., 2025; Li et al., 2022; Lúcio et al., 2024). Nevertheless, most clinical studies have been limited by relatively small sample sizes, short intervention periods, and heterogeneous findings. Moreover, current evidence has not consistently demonstrated improvements in hard renal outcomes, such as the rate of estimated glomerular filtration rate (eGFR) decline, initiation of dialysis, or progression to kidney failure (Nogueira-Rio et al., 2025; Biruete et al., 2021; Lúcio et al., 2024). At present, these interventions are therefore more appropriately considered adjunctive strategies for improving the metabolic milieu rather than disease-modifying therapies for CKD.

Direct supplementation with SCFAs has demonstrated renoprotective effects in experimental models. Butyrate, propionate, and other SCFAs attenuate oxidative stress, inflammation, and fibrosis in DKD and other models of experimental kidney injury through mechanisms involving GPR43/GPR109A signaling, histone modification, mitochondrial metabolism, and immunoregulation (Corte-Iglesias et al., 2024; Ma and Wang, 2022; Ye et al., 2024; Li et al., 2020; Huang et al., 2020; Li et al., 2023). However, clinical trials evaluating direct SCFA supplementation in patients with kidney disease are currently lacking. The optimal dosage, delivery strategy, tissue exposure, and long-term safety profile also remain undefined, indicating that the clinical translation of this approach is still at an early stage.

Modulation of tryptophan metabolism likewise exhibits marked ligand- and disease-specific effects. Indole-3-aldehyde (I3A) has been shown to alleviate cisplatin-induced AKI by improving mitochondrial function, whereas pathological activation of AHR has been implicated in membranous nephropathy, renal aging, and fibrotic remodeling (Yuan et al., 2024; Wang Y. N. et al., 2023; Xie et al., 2024; Wang Y. N. et al., 2025). Accordingly, a more rational therapeutic strategy may be to distinguish protective from pathogenic AHR ligands and selectively modulate aberrant AHR signaling rather than broadly activating or inhibiting this pathway.

Interventions targeting FXR and TGR5 have improved lipid metabolism, mitochondrial function, and fibrotic phenotypes in experimental models of diabetes- and obesity-associated kidney injury (Zhou et al., 2025; Yang et al., 2024; Lin et al., 2023; Wang et al., 2018). However, kidney disease-specific clinical trials remain scarce. Moreover, part of the mechanistic evidence supporting the anti-inflammatory and antifibrotic effects of FXR/TGR5 signaling has been derived from studies of liver disease (Sun et al., 2026). These findings should therefore be regarded as indirect mechanistic support rather than direct evidence of clinical efficacy in kidney diseases.

### 5.2 Reducing the burden of pathogenic metabolites and blocking injurious signaling pathways

Elevated levels of uremia-associated metabolites and selected proinflammatory metabolic mediators are common in CKD. However, their circulating concentrations are determined not only by microbial production but also by host metabolism, dietary intake, pharmacological exposure, protein binding, and renal clearance (Delrue et al., 2026). Consequently, increased metabolite levels should not be interpreted as independent pathogenic drivers, nor should reductions in their circulating concentrations necessarily be expected to translate into renal functional benefit.

AST-120 adsorbs precursors of uremic toxins within the intestinal lumen and thereby reduces the burden of selected IS-related metabolites. Clinical studies have shown that AST-120 can influence gut microbial composition and alter the profiles of short- and medium-chain fatty acids in patients with advanced CKD. Experimental studies have further suggested beneficial effects on the intestinal environment, renal oxygenation, and kidney injury phenotypes (Hsu et al., 2022; Yoshifuji et al., 2018; Sivertsson et al., 2023). Nevertheless, clinical trials have not consistently demonstrated improvements in long-term hard renal outcomes. AST-120 is therefore more appropriately described as an intervention with metabolic and experimental renoprotective effects rather than as an established renoprotective therapy.

Low-protein diets supplemented with ketoacids reduce the availability of protein fermentation substrates and can influence microbial metabolism as well as the concentrations of protein-bound uremic toxins (Rocchetti et al., 2021; Wu et al., 2020; Lohia et al., 2022; Chang et al., 2023). These strategies have been evaluated in patients with CKD, but the observed clinical benefits cannot be attributed solely to changes in microbial metabolism. Their implementation also requires careful balancing of protein restriction against the risks of malnutrition, sarcopenia, and poor dietary adherence. Accordingly, dietary interventions should be individualized according to the stage of kidney disease and nutritional status rather than being guided exclusively by the goal of lowering uremic toxin levels.

Direct inhibition of microbial trimethylamine (TMA) production represents a more targeted metabolic intervention. Inhibitors of the CutC/D pathway reduce TMAO generation and have attenuated tubulointerstitial fibrosis and renal dysfunction in murine models of CKD (Gupta et al., 2020; Cheng et al., 2025; Zhang et al., 2021). However, clinical evidence remains unavailable, and it is unclear whether long-term suppression of a single microbial metabolic pathway may induce ecological compensation, substrate redistribution, or other unintended metabolic consequences.

Blocking metabolite-associated receptors or downstream signaling pathways has also emerged as a potential therapeutic strategy. Experimental animal and cellular studies indicate that IS-AHR signaling contributes to renal aging, inflammation, and fibrosis, whereas SUCNR1 signaling participates in tubular phenotypic alterations under hyperglycemic conditions (Xie et al., 2024; Zhang et al., 2024; Wang Y. N. et al., 2025; Pu et al., 2024). However, these receptors also play essential roles in physiological immune regulation and metabolic homeostasis, raising concerns that complete inhibition could produce undesirable off-target effects. Current evidence therefore favors selective modulation of pathological signaling within specific disease contexts and target-cell populations rather than broad receptor blockade.

### 5.3 Remodeling microbial metabolic function

Compared with directly supplementing or eliminating individual metabolites, remodeling the overall metabolic function of the gut microbiota has the potential to simultaneously influence intestinal barrier integrity, substrate utilization, and multiple metabolic outputs. However, this strategy is also associated with greater interindividual variability and ecological complexity.

Studies of selected probiotics and synbiotics have reported reductions in certain uremic toxins or inflammatory biomarkers, although substantial heterogeneity exists with respect to microbial strains, dosage, treatment duration, and baseline microbial composition (Mitrović et al., 2023; Kuskunov et al., 2023; Hua et al., 2026; Jang et al., 2026). To date, small clinical studies have not provided consistent evidence for improvements in renal function. Accordingly, these interventions should not simply be described as increasing “beneficial bacteria.” Instead, future studies should more precisely define strain-specific functions, associated metabolic outputs, and the patient populations most likely to benefit.

Fecal microbiota transplantation (FMT) simultaneously modifies microbial composition and multiple metabolic pathways. Experimental studies in CKD have suggested that FMT may reduce PCS levels, ameliorate microbial dysbiosis, and attenuate selected manifestations of renal injury. However, findings remain inconsistent, and some models have shown little or no improvement in renal function (Barba et al., 2020; Arteaga-Muller et al., 2024; Liu X. et al., 2022). Furthermore, adequately powered clinical trials in CKD and other kidney diseases are still lacking. Donor selection, pathogen transmission, long-term ecological stability, and protocol standardization remain major challenges. Consequently, FMT should currently be regarded as a preclinical or early translational strategy rather than an established therapeutic option for kidney diseases.

Engineered microbial therapeutics offer the possibility of more precise functional regulation than conventional probiotics by selectively consuming uremic toxin precursors, degrading pathogenic metabolites, or enhancing the production of protective metabolites. Experimental studies have shown that approaches such as SynCKD and engineered *Escherichia coli* can reduce uremic toxin generation and improve selected indices of kidney injury (Beau et al., 2025; Lambert et al., 2023; Lubkowicz et al., 2024). However, long-term colonization, genetic stability, biosafety, environmental containment, and clinical efficacy remain to be established. These approaches therefore remain at the preclinical stage.

Bacteriophage therapy provides another strategy for selectively eliminating target bacterial populations and thereby reshaping microbial metabolic function. At present, however, evidence supporting precise modulation of the gut microbiota and metabolome is derived predominantly from experimental models outside the field of nephrology (Zhang et al., 2025; Liu C. et al., 2025). Its application in kidney diseases should therefore be considered proof of concept rather than evidence of clinical efficacy or safety in CKD.

### 5.4 Precision metabolic-microbiota interventions and future translational directions

Kidney diseases are characterized by marked heterogeneity in etiology, stage of renal dysfunction, and metabolic phenotype. Future therapeutic strategies will therefore likely need to shift from empirical modulation of the gut microbiota toward stratified interventions guided by metabolic-immune characteristics. Metagenomics, metabolomics, single-cell sequencing, and spatial omics provide powerful tools for identifying metabolic abnormalities, cellular interaction networks, and candidate therapeutic targets (Abedini et al., 2024; Liu H. et al., 2022). At present, however, these technologies are used primarily for mechanistic investigation and risk stratification rather than for clinically validated treatment selection.

Collectively, the available evidence indicates that dietary fiber, prebiotics, probiotics or synbiotics, AST-120, and low-protein or ketoacid-supplemented diets have accumulated a degree of support from human studies. Nevertheless, most investigations have focused on changes in microbial composition, circulating metabolites, nutritional status, or inflammatory biomarkers, whereas evidence for improvements in hard renal outcomes remains limited or inconsistent (Nogueira-Rio et al., 2025; Biruete et al., 2021; Hsu et al., 2022; Rocchetti et al., 2021; Wu et al., 2020; Mitrović et al., 2023; Kuskunov et al., 2023; Lúcio et al., 2024; Chang et al., 2023; Hua et al., 2026; Jang et al., 2026). By contrast, SCFA supplementation, modulation of the tryptophan-AHR axis, activation of FXR/TGR5 signaling, inhibition of TMA production, and regulation of SUCNR1 are supported predominantly by experimental animal and *in vitro* studies (Li et al., 2020; Huang et al., 2020; Yuan et al., 2024; Wang Y. N. et al., 2023; Lin et al., 2023; Wang et al., 2018; Gupta et al., 2020; Cheng et al., 2025; Xie et al., 2024; Zhang et al., 2024; Li et al., 2023; Zhang et al., 2021; Wang Y. N. et al., 2025; Pu et al., 2024). FMT, engineered microbial therapeutics, and bacteriophage-based approaches remain at the stage of animal studies, preclinical development, or proof-of-concept investigations outside kidney disease (Barba et al., 2020; Arteaga-Muller et al., 2024; Beau et al., 2025; Lambert et al., 2023; Zhang et al., 2025; Liu C. et al., 2025; Liu X. et al., 2022; Lubkowicz et al., 2024).

Accordingly, the current evidence does not support the development of definitive patient stratification algorithms or metabolite-guided therapeutic decision frameworks. In CKD, future studies should prioritize the relationships among uremic toxin burden, renal clearance, intestinal barrier integrity, and nutritional status. Investigations in DKD should additionally account for hyperglycemia, lipotoxicity, and systemic metabolic dysfunction, whereas direct mechanistic and clinical evidence supporting microbiota-targeted metabolic interventions remains particularly limited in IgAN and LN. Future progress will depend on disease-specific cohorts, mechanism-based clinical trials, standardized metabolite profiling, and kidney-specific clinical endpoints to determine whether improvements in microbial or metabolic signatures can ultimately be translated into sustained preservation of renal function.

## 6 Summary and perspectives

In recent years, microbiota-associated metabolic networks have emerged as an important conceptual framework linking the intestinal ecosystem, host metabolic status, and renal immune responses. Rather than viewing individual metabolites as isolated effector molecules, current evidence increasingly supports the concept that these networks comprise direct microbial metabolites, host-microbiota co-metabolites, and host-derived immunometabolic mediators influenced by the gut microbiota. Although these metabolite classes differ in their biological origins and functional properties, they ultimately converge on several shared pathological processes, including maintenance of intestinal barrier homeostasis, stress responses and repair of renal tubular epithelial cells, immune-cell reprogramming, persistent inflammatory amplification, and tubulointerstitial fibrosis. Through bidirectional gut-kidney feedback, these interconnected processes may continuously influence disease progression. Accordingly, microbiota-associated metabolic networks may represent not only accompanying metabolic alterations in kidney disease but also an important regulatory layer linking intestinal dysbiosis, host metabolic dysfunction, and renal immune remodeling. Importantly, this shared metabolic-immune framework should not be interpreted as evidence that all kidney diseases follow identical molecular mechanisms. Rather, different kidney diseases appear to share common immunopathological modules while exhibiting distinct dominant metabolic signals, target-cell populations, and regulatory pathways according to disease context.

Despite substantial progress, the current evidence remains markedly uneven across different kidney diseases. The strongest mechanistic evidence has been generated in AKI, CKD, DKD, uremic toxin accumulation, and experimental renal fibrosis, where SCFAs, selected tryptophan-derived metabolites, IS, PCS, and TMAO have been investigated in experimental models, cellular systems, and selected human studies and have demonstrated biologically relevant roles in regulating renal inflammation, immunometabolism, and fibrosis. By contrast, evidence in immune-mediated kidney diseases such as IgAN and LN remains focused largely on associations among gut microbial composition, metabolic profiles, and immune phenotypes, whereas direct mechanistic studies linking specific microbiota-associated metabolites to renal immune regulation are still limited. Furthermore, human observational studies, experimental animal models, *in vitro* investigations, and mechanistic evidence derived from other inflammatory diseases support different levels of inference. In particular, circulating concentrations of metabolites such as IS, PCS, and TMAO are influenced not only by microbial metabolism but also by declining renal function, impaired metabolite clearance, dietary factors, and therapeutic interventions. These metabolites should therefore currently be regarded as integral components of metabolic-immune feedback during disease progression rather than being interpreted as independent pathogenic drivers. Future investigations should more clearly distinguish the interpretive boundaries of different evidence types and avoid directly extrapolating mechanisms identified in non-renal diseases or observational associations into established causal pathways in kidney diseases.

From a translational perspective, dietary fiber, prebiotics, probiotics or synbiotics, AST-120, and low-protein or ketoacid-supplemented diets have accumulated a degree of support from human studies. However, most clinical investigations have focused on microbial composition, metabolite concentrations, nutritional status, or inflammatory biomarkers, whereas evidence for sustained improvements in long-term renal outcomes remains limited or inconsistent. By contrast, SCFA supplementation, modulation of AHR or SUCNR1 signaling, activation of FXR/TGR5, and inhibition of TMA production remain supported primarily by experimental animal and cellular studies. FMT, engineered microbial therapeutics, and bacteriophage-based strategies are still largely confined to preclinical or proof-of-concept investigations. Accordingly, no single microbiota-targeted metabolic intervention can currently be regarded as an established therapeutic strategy applicable across different kidney diseases.

Future research should move beyond descriptive analyses of microbial composition or individual metabolites toward a more comprehensive understanding of the dynamic functional states of microbiota-associated metabolic networks and their spatiotemporal regulation across diverse kidney disease contexts. Integration of metagenomics, metabolomics, single-cell sequencing, spatial omics, stable isotope tracing, and kidney organoid technologies is expected to clarify the biological origins of individual metabolic signals, identify their critical cellular targets, and define their dynamic interactions within the renal microenvironment. Such advances may facilitate the development of an integrated framework linking microbiota-associated metabolic signaling, host immune remodeling, and kidney disease outcomes. At the same time, longitudinal cohort studies, genetic approaches, and targeted functional validation will be essential for distinguishing disease-driving metabolic pathways from secondary alterations associated with declining renal function and for defining the relationships between shared metabolic-immune modules and disease-specific regulatory networks across different kidney diseases. As precision nutrition, metabolite-targeted therapies, engineered microbial therapeutics, and personalized microbiome-based interventions continue to evolve, modulation of renal immune microenvironments through microbiota-associated metabolic networks may provide new opportunities for patient stratification, therapeutic response prediction, and precision intervention in kidney diseases.
